# Food regulation and policing: innovative technology to close the regulatory gap in Australia

**DOI:** 10.1007/s00003-022-01372-2

**Published:** 2022-03-05

**Authors:** Jade Lindley

**Affiliations:** The University of Western Australia, Crawley, WA Australia

**Keywords:** Food fraud, Regulation, Traceability, Regulatory pluralism

## Abstract

Internationally, food regulations are centred on human health and safety to prevent health crises. In Australia, regulatory control over the health and safety of humans is sound, however from a criminological perspective, control over fraudulent activities within food supply chains lack. Food fraud knows no geographical boundaries and has endless reach, therefore should be prioritised by policymakers, regulators and law enforcement. Australia’s reputation for high-quality food is important domestically, but also for establishing and maintaining trust in international food trade relationships, therefore lack of enforcement over food could damage ‘Brand Australia’. Given the food industry’s vested interest in maintaining this reputation, it must also play a role to protect it. This research reviews regulatory landscape against food fraud in Australia and then, questions whether coupling informal controls to support existing formal regulatory controls may be the most appropriate and holistic way forward to protect the industry and consumers. It tests a regulatory pluralism framework to determine whether it can logically organize informal, innovative responses to contribute cohesively alongside formal controls at various points along the supply chain to prevent food fraud. Finally, it considers available informal, innovative technologies to: enhance testing regimes; prevent product and label tampering; and trace food supply chains adopted internationally show positive progress in responding to increasingly sophisticated and organized global food fraud. The research concludes adopting a regulatory pluralism framework, coupling existing regulatory controls and innovative technology could enhance and strengthen Australia’s regulatory response to fraud within its food industry.

## Introduction

Food fraud costs the global food industry approximately US$49 B annually (Williams 2018). Spink (2011) and others suggest food fraud involves intentional deception for profit including food mislabeling, adulterating, misrepresenting country of origin, weight and/or nutrition, and repackaging. A borderless crime, for centuries food fraudsters look for opportunities to infiltrate supply chains to yield profits comparable to cocaine trafficking, with lower risk (Mueller 2007). Food fraud often occurs alongside other enabling crimes such as corruption, document fraud and smuggling to evade detection (United States Pharmacopeial Food Safety and Integrity Solutions 2016; Interpol 2018). Thus, the potential harm is significant.

Australia, like most other importing and exporting nations, experiences some food fraud. Since its creation, the European Commission’s Rapid Alert System for Food and Feed (RASFF) System recorded various forms of fraud involving Australian produce (European Commission 2020). Despite low incidences reported through the RASFF, regulation and enforcement at Australia’s borders and within Australian food industries remains critical to protect Australian and foreign consumers.

Regulatory responses to address food fraud globally and in Australia are often inadequate, though national food laws and their enforcement are pivotal in fraud control (Spink et al. 2016). Recent examples in Australia have revealed vulnerabilities due to inadequate laws, e.g. cooked seafood (Lindley 2021b); insufficient testing, e.g. imported honey (Zhou et al. 2018) and oregano (Choice 2016); and unclear definitions enabling food fraud (Lindley 2021a). Limited penalties and patchy regulatory control over food fraud enables criminal syndicates to thrive. Outdated and ill-equipped regulations are aspects of the problem.

On top of regulatory challenges, food fraud is difficult to adequately police. As food fraud is transnational, regulatory compliance and enforcement inconsistencies limit the ability to effectively police between borders (Curll 2015). Limited by inconsistencies in defining ‘food fraud’, it remains a food safety risk rather than a policing issue and coordination between food and consumer regulators remains fractured (Curll 2015). As such, informal and non-traditional approaches to respond to fraud must be explored.

Globally, technology is constantly being developed to enhance the agri-food sector and close the regulatory gap on fraud, some of which has been adopted in Australia (Pandian 2020; Yadav et al. 2020). For example, research considers the viability of various technologies in Australian seafood (Australian Government 2020; Bird 2020), beef (Marshall 2018; Condon 2019; Futures Centre 2020), and wine industries (Liu et al. 2006; Pereira et al. 2018). These emerging technologies provide useful regulatory support however, they are voluntary, and industry rather than government-led. Without clear policy directives to adopt such technologies, loopholes enabling fraudulent activity remain leaving some industries or foods more exposed than others.

In order to determine the most appropriate approach to control food fraud in Australia, it is necessary to first review overarching regulatory frameworks (Sect. 2). This review reveals vulnerabilities to fraudulent activities. Non-traditional, informal and innovative complimentary measures may be appropriate in lieu of amending existing or introducing new regulatory tools to collectively protect against food fraud. In applying a collective approach to respond to food fraud, it may be useful to draw on a regulatory pluralism framework to support formal regulation (Sect. 3). As such, this research tests whether a regulatory pluralism approach could be suitable to address food fraud. An extensive academic literature review reveals technology already available and working in unison with regulatory responses could provide greater support to protect the Australian brand, food industries, and consumers (Sect. 4).

## Regulating and policing food fraud

### Regulating food fraud in Australia

Globally, regulations exist to protect consumers and the food industry. Comparing traceability regulations across OEDC countries, Australia and New Zealand both received overall world ranking scores of *average*, while European Union and pan-European countries scored the highest *superior* rank (Charlebois et al. 2014). Though across the board, food regulations are concerned primarily with food safety rather than the criminal behaviours, there is a critical need for a criminological food fraud perspective to understand acts and methods of food-related crimes and to develop efficient and effective countermeasures and control systems (see for example Spink and Moyer 2011; Lord et al. 2017; van Ruth et al. 2017; Esteki et al. 2019; Spink 2019; Borraz et al. 2020).

Internationally, a partnership exists between the World Health Organization (WHO) and the Food and Agriculture Organization of the United Nations (FAO) relating to food safety, and together they established the *Codex Alimentarius.* The Codex provides for its 188 members a means of standardizing quality of food trade (Food and Agriculture Organization of the United Nations 2020). The Codex significantly influences global food laws. This extends to Australia and New Zealand, two countries that merged their food regulatory control.

The cooperative Food Treaty between Australia and New Zealand reduces “unnecessary barriers to trade” (Food Regulation 2019) and enables the Australian and New Zealand governments (state and federal) to harmonize food safety and ensure it is efficiently controlled throughout the supply chain. Building on the Food Treaty is the operational Australia New Zealand Food Standards Code. Each jurisdiction must comply with the Treaty and Code, in addition to any locally adopted regulations overlaying further compliance.

Despite local differences, human safety is the primary concern of food labelling, with little focus on the criminal aspect. Australian and New Zealand food labeling is guided by three overarching priorities (Food Regulation 2016):

1. food safety, relating to immediate health threats;
2. preventative health, relating to chronic disease; and
3. Consumer values, dealt with by consumer protection law rather than food regulation.

Mislabeling is only one element of illegal activities that sits under the umbrella of the food fraud definition.

Local and imported food consumed in Australia is strictly regulated. Food legislation is administered at the state level mirrors overarching federal food legislation. Food fraud affecting Australian consumers is dealt with in two ways: administratively at the state level (via the local *Food Acts*) and criminally at the state and/or federal level via criminal code provisions for fraud (Australian Government 1995). Most food offences are dealt with administratively and the approach to diversion and penalties involves a progression from warnings and reparation, followed by increased scrutiny and prohibition, and finally prosecution is used as a last resort (Australian Competition and Consumer Commision 2016). Penalty severity is applied accordingly.

Penalties under a charge of fraud would be significantly more severe. Intentional food contamination attracts harsher penalties since the 2018 Australian strawberry scandal, the maximum penalty increasing from 10 to 15 years of incarceration (Australian Government 2018). Organised food criminals would expect harsher penalties, compared to food adulterators or those who intentionally mislabel. Most often, cases are investigated by the Australian Competition and Consumer Commission (ACCC), an independent statutory authority within the Australian government. Administrative sanctions such as infringements relating to food harms can be applied to individuals or corporations, adjusted to reflect the harm (Australian Competition and Consumer Commision 2020). Based on publicly available information, food fraud is rarely pursued criminally, indicative of a lack of criminal interception rather than an absence of crime.

Collectively, laws and standards in Australia amount to adequate control over the food industry. However, it appears food regulators tend to focus on direct safety risks, rather than the criminal aspect. The system relies on consumers or public health campaigners to make food complaints to the ACCC before action is taken or an investigation launched (Bedo 2018) and even when food fraud is investigated, many consumers would be unaware.

### Policing food fraud: lessons from abroad

Internationally, Interpol is tasked with preventing and intercepting food fraud and related crimes. Jointly coordinated with Europol, Operation OPSON (which translates to *food* in ancient Greek) began in 2011 in 10 mainly European countries. Now it operates in 80 countries, including Australia (Europol 2020a). Supported by domestic police forces, OPSON seized more than 16,000 t and 33 million litres of fraudulent food and drink estimated at US $117 M and made 672 arrests having conducted more than 50,000 regulatory checks (Europol 2017, 2020a). OPSON seized expired food or food with altered expiry dates, suggesting disrupted food supply chains during COVID-19 were targeted (Europol 2020b). Criminal resilience and adaptability during a pandemic indicate the need for greater policing sophistication to intercept food fraud.

Food safety is a national priority, including in Australia. Mostly focused on minimizing allergen and food poisoning risk, foods are tested and checked to ensure they meet international, industry and domestic standards for human consumption. Law enforcement occasionally has dedicated food enforcement and investigation units, working with OPSON. Following the horsemeat scandal of 2013, the United Kingdom established the National Food Crime Unit (Food Standards Agency 2020). The Unit has dedicated law enforcement functionality benefiting industry and the public (Food Standards Agency 2020). Dedicated food fraud teams in Australian law enforcement would no doubt increase consumer confidence in response to food fraud.

The need of dedicated food policing units is debatable, though when policing any transnational organized crime, in-depth knowledge of common activities, players and peripheral crimes can help close down criminal operations. For example, two methods of food fraud are common: making a product appear of higher value, or supplying food or ingredients unfit for consumption (Davies 2020). Policing research indicates specialized officers within dedicated units can achieve swifter outcomes (see for example Bayley and Weisburd 2009; van Staden and Lawrence 2010; Button et al. 2014). Dedicated food police would better understand vulnerable products and the specific *modus operandi* of criminals, such as the fraudulent insertion of tiny lead slivers into *Matsutake* mushrooms to inflate their weight and value (priced around US$ 1000 per lbs) (Shapiro 1989).

Human harm is not often the focus or outcome of intentional food fraud; harm caused by food goes beyond the physical harm (Manning and Soon 2016). Proceeds from food fraud may fund other serious crimes such as human and drug trafficking (Jacobs 2014), providing strong incentive to quell food fraud. As such, pluralistic responses, combining traditional policing and non-traditional, innovative and industry-led measures are critical to ensure law enforcement operates similarly sophisticated responses to food-related crimes.

## Regulatory pluralisms: a patchwork of regulators

Food fraud responses require support beyond regulatory controls. This research tests whether a regulatory pluralism approach may more effectively bridge legal divides, otherwise insurmountable to traditional law enforcers. Existing technology to test food safety and regulations on food safety, coupled with emerging non-traditional industry-led technologies to detect fraud, could holistically respond (Spink and Moyer 2011). Policy to mandate technology roll-out can maximise its usefulness, shifting the cost benefit balance in favour of law enforcement in response to food fraud.

**Fig. 1:**
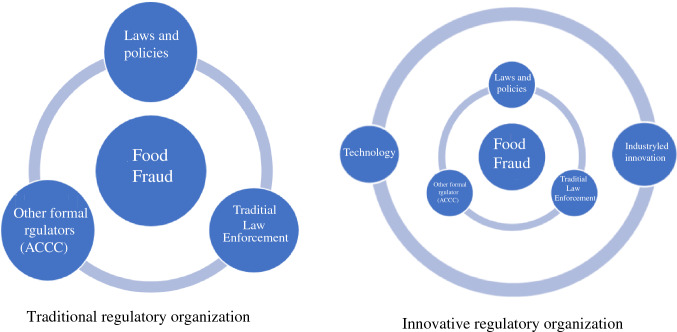
Traditional regulatory organization and innovative regulatory organization. Traditional regulation of food fraud in Australia (left) compared to a regulatory pluralism approach (right). Traditional regulation has a clear, formal regulatory response available to respond to food fraud, whereby laws and policies, traditional law enforcement and other formal regulators such as the ACCC cooperate to respond. An innovative regulatory response involves an informal layer to support the traditional actors. An innovative regulatory pluralism response involving industry-led regulators and innovative technology, can collectively and more holistically prevent food fraud

Regulatory pluralism provides a vehicle to close criminal loopholes and enhance industry-led vigilance, using both formal and informal regulators. Regulatory pluralism theorizes a single regulator or *actor* may be less effective than a collaborative regulatory partnerships (Grabosky 1995). It suggests the traditional state-led regulatory monopoly is instead supported by a patchwork of alternate formal and informal actors. Regulatory pluralism is useful in environments with multiple jurisdictions, stakeholders and regulatory regimes (and loopholes), creating layers of complexity (Gunningham and Grabosky 1998), as is the case with food fraud and other complex crimes (see for example Lindley and Techera 2017; Lindley 2018, 2019). In practical use, informal food regulators such as certification organizations (e.g. Marine Stewardship Council or Fairtrade International), apply a threshold that may transcend borders, connecting industry with importing and exporting nations. While its motivations are to ensure the product meets its own certification requirements, it overlaps with formally regulated international and national standards (Fig. 1).

## A regulatory pluralism response: coupling regulations and technology to address food fraud

Technology currently supports the control of food fraud in many ways, including prevention and detection. Scientific testing is essential to uncover adulteration and advancements in technology have expanded its application, ease of use, and reliability. Implementing emerging technology is key to detect and prevent mislabeling, which can improve food provenance, whether for highly priced, or common everyday staple foods. Traceability tools increases trust in the brand leading to increased demand and value. While investment in technology may be costly and arduous, the potential benefits may outweigh the risks. Drawing on regulatory pluralism, this section explores the use of emerging technologies to support traditional regulatory systems to detect and prevent food fraud. While technology evolves rapidly, those outlined below show promise within the food sector at the time of writing.

### Interception: enhanced testing technologies to detect adulterated food

Scientific testing can rapidly confirm food safety, detect food fraud and verify authenticity of labeled produce, essential for transparent supply chains. Traditional approaches, such as sensory, chemical, chromatographic, molecular, and protein-based techniques, among others, are used to identify animal species, production methods, provenance, and processing of food products (Esteki et al. 2019; Hassoun et al. 2020). These traditional tests may be destructive to food, time-consuming, and require laboratory-scale testing facilities, which are not always available (Hassoun et al. 2020). Outdated tests may also be easily beaten by sophisticated criminals (see e.g. Hatch 2018; Zhou et al. 2018). Instead, emerging methods are available drawing primarily on spectroscopy that overcome many of these limitations (Xiong et al. 2016b; Esteki et al. 2019; Hassoun et al. 2020). Each method has advantages and disadvantages, depending on the sample and application (Esteki et al. 2019).

Beyond spectroscopy, research confirms the effectiveness of DNA barcoding as an food authentication tool (Wallace et al. 2012; Xiong et al. 2016b). Drawing on DNA markers, modernized technologies may be used to verify origin, accurate enough to verify the specific farm of origin by testing elements from plants animals ate, including whether the animal was raised free-range (Tickle 2013). DNA barcoding can authenticate plant and animal origin foods therefore has widespread applicability, used in Ireland to expose the 2013 European horsemeat scandal (Reilly 2018).

While conventional tests yield useful results, tests using emerging technology may provide higher quality results. Some forms of testing may be expensive, inaccessible and advances in technology may be challenging for government-led testing facilities to justify maintaining (Zhou et al. 2018). As such, coordinated links between government and industry can bridge the divide and ensure tests conducted produce reliable results. Samples held overtime could be easily re-tested years later once technology improves (National Criminal Justice Reference Service 2002), though the urgency of results to circumvent the supply chain is time critical. The Australia government, among others must mandate innovative testing, and when carried out by industry, it contributes to a broader patchwork of formal and informal regulation, aligned with regulatory pluralism.

### Prevention: using technology to safeguard against mislabeling food

The role of food labeling is to inform the consumer to prevent critical human health harms, including allergic reactions; provide health information to assist the consumer make informed decisions about what they consume; and allow consumers to decide whether certain food aligns with their values and ethics (Food Regulation 2016). The importance of the accuracy and reliability of information printed on these labels, therefore cannot be overstated; however, food fraud undermines labeling for profit by *mis*labeling.

Mislabeling food occurs due to lack of transparency in the food supply chain. Opportunist criminals take advantage of often transnational supply chains through enabling crimes, such as corruption and document fraud, to gain a profit. Due to these enabling crimes, current traceability systems that may be monopolistic, asymmetric and opaque lend themselves to fraud and potentially, reduced trust in the brand or product (Varghese et al. 2020). The net result is legitimate industry and consumers are unknowingly victims of food fraud. To ensure criminals are unable to circumvent the systems in place, greater protections are needed, such as non-traditional, sophisticated, and potentially emerging technologically-based methods, which may be the most appropriate means to secure the supply chain.

There is no silver bullet option to control food fraud. Rather, endless options exist that may suit the particular industry and/or product. As with all technology however, there are potential barriers and challenges, such as cost of implementation, maintenance and upgrades; reliability; access in remote locations, such as on the high seas and remote farms; user-friendliness, access to, and availability of user training and support; and guarantee the technology solution is fit for purpose. Despite these barriers and challenges, the potential protections afforded by these solutions could outweigh the risk. Some examples are explored in the following section.

### Blockchain

Blockchain is one of the most exciting technological advancements in food fraud suppression. It digitizes secure transactions or *blocks* at every point along the supply chain and is decentralized allowing anyone with access to that specific blockchain ledger to access it, eliminating the need for intermediaries (Dawson 2018; Braeken et al. 2020; Yadav et al. 2020). Each block is encrypted with a unique, non-manipulable identifier and therefore completely transparent and traceable, starting with the source, assuming the producer is not engaging in fraud. Blockchain operates outside the food industry, for example in gem and textiles trade, though the lower cost for unit may have been seen as an initial restrictor for use with food tracing (Dawson 2018; Cui and Leonas 2020).

At relatively low cost, blockchain offers the ability to track in real time, removes the need for time consuming (potentially fraudulent) document processing, while realising cost saving efficiency, increases its usefulness (Scattergood 2018; Antonucci et al. 2019; Keogh et al. 2020; Marecki and Wójcik-Czerniawska 2020; Pérez et al. 2020). From a perspective of regulatory pluralism, blockchain provides a platform upon which all actors within the supply chain are legitimized. Through the process of transnational transportation, ordinarily countless supply chain vulnerabilities would be encountered but blockchain closes the loop on potential fraudulent activities.

Blockchain has been utilized in many high-risk foods and industries. For example, research considers its application within the Thai and Chinese fishing industries (Xiong et al. 2016a, b; Tsolakisa et al. 2020); to enable livestock disease warning, authenticating food supply chain, and tracking sources of contamination in the production cycle (Yang et al. 2020); to monitor fast food delivery to minimize risk of food poisoning (Singh et al. 2021); community-based farmer-to-consumer traceability, removing intermediaries and potentially any price injustices (Jaiyen et al. 2020); and to trace beef provenance from farm to fork via blockchain-enabled beef passports issued upon birth registration (Rymer and Freeman 2016), then sold via the new *BEEF Token* cryptocurrency, via the blockchain *BeefLedger* (Condon 2019). These examples enable greater producer autonomy and consumer confidence in managing agri-food in more efficient and optimized ways, aligned with the regulatory pluralism principle (Torky and Hassanein 2020).

Lack of buy-in from all points in the supply chain will disadvantage the usefulness of blockchain (Scattergood 2018; Fan et al. 2020). Ocean freight is still the most common form of food transport, and as such, the effectiveness of blockchain also depends on uptake for all involved points along the shipping supply chain.

Predictions suggest eventually, all transnational trade will be transacted on blockchain platforms due to its impenetrable application, despite some present limitations on scalability and network capacity (Scattergood 2018; Stipic 2020). Vast literature studies by Goundar et al. (2020) and Rejeb et al. (2020) found overwhelmingly there is support for introducing blockchain technology in food supply chains. Removing anonymity in food supply chains, blockchain technology can support a safer, smarter, and more sustainable food supply (Varghese et al. 2020), however, the cost of adopting blockchain technology will no doubt be passed along to the consumer, therefore awareness raising communication must be made available (Colomberotto 2020; Fan et al. 2020). Government subsidization of these costs could ensure implementation while protecting the consumer from paying more.

### Individual product labeling

Given the potential for packaging to be fraudulently mislabeled along the supply chain, labeling the individual product is emerging as a viable method. Alternate labeling can occur on the product itself or on the packaging, both of which may be invisible to the naked eye, and more challenging to manipulate. Despite increased cost in applying these additional layers of protection, customer satisfaction and brand trust will no doubt increase.

Fraudulently branded Australian beef costs approximately AUD$ 2 B annually and as such a loss in reputation and international customer-base may be irreparable (Marshall 2018). In addition to blockchain, other technologies are being tested to fill the regulatory gap, such as edible fingerprinting built in partnership by PwC, Google and Japan’s Nitto Denko (Marshall 2018). The process involves spraying the meat with a natural nano-scale silicon dioxide particle, or edible fingerprint, similar to traceability signatures already used to guarantee the authenticity of pharmaceutical products (Marshall 2018; Futures Centre 2020). This edible fingerprint spray is applied as the meat is packed in Australia and can survive temperatures from − 20 to 400 °C enabling authentication even after the cooking process, enabling testing at any stage between processing and consumption (Futures Centre 2020).

Another form of product labeling, albeit more overt, has been adopted by the Tasmanian Oyster Co. Having received government and industry grants to develop a purpose built etching robot, the producer-driven initiative laser etches their logo into the oyster shells to satisfy provenance and distinguish the product from others on the market (Advanced Manufacturing Growth Centre Ltd 2020; Bird 2020). Despite the resulting increased production cost, the company intends to increase its export quantity, and at a higher price per unit (Bird 2020). This innovative approach enables increased confidence in the product.

The external food label presents great opportunities to utilize anti-fraud technology. For example, luxury foods in particular are looking to anti-fraud technologies including invisible particles mixed into food label printing ink and plastic packaging or caps detectable only by the dedicated scanners (NanoMatriX 2020). Encoded identification tags and quick response (QR) codes can be loaded with information to prevent tampering or counterfeiting and provide additional information about the product, including provenance (Xiong et al. 2016a; Reilly 2018; NanoMatriX 2020). Expanding the remit of existing, well-tested technology may minimize opportunity to commit food fraud, should criminals access dedicated scanners and mimic ink particles on fraudulent food labels, the technology can work against the initial aim. Sophisticated regulatory responses must stay abreast of emerging technology to ensure food supply chains are adequately tested and labeling methods are traceable and transparent.

### Industry-led initiatives

At the industry level, control over standards and enforcing those standards is paramount. Though systems have been in place in the past, comprehensive food fraud management systems are new for the food industry (Hoffman 2020). “The nature of food fraud combined with differences in data tracking systems make it almost impossible to reconcile the data among the various systems” (Everstine 2019). Various food industries are leading by implementing transparent resilience modelling and benchmarking safety (such as the international Global Food Safety Initiative) to avoid preventable supply chain infiltration, which could be costly to the brand (Havinga and Verbruggen 2017). By increasing the maturity, or resilience of the system, enables producers to minimizing fraud, risk of product recalls, loss of contracts and other lost revenue.

Lacking government mandate led some Australian industries and producers to explore options to circumvent fraud. For example, the Australian egg industry commissioned research to review its existing egg traceability systems. Australian consumers have longed questioned the truth in free-range and cage-free eggs, compared to barn laid—despite the price premium at the checkout. The report found “traceability is challenging as the egg supply chain is quite fragmented” (Szabo et al. 2020). Similarities can be drawn to other industries, such as seafood. While industry-led initiatives align with a regulatory pluralism approach, implementation of industry self-regulation frameworks requires policy directive to ensure compliance among all industry participants ensuring consumers are able to trust an industry, not just a brand.

## Conclusion

In Australia, food is well regulated and has a strong reputation of safety, increasing trust among local and international consumers alike. However, brand Australia cannot rest on its laurels; rather it must maintain, if not exceed the international standard in regulating, policing and testing.

Technology is already an essential element to detect and prevent food fraud, though it must be modernized to stay abreast of criminal sophistication. Scientific testing is ever-developing and improvements in existing, as well as new technologies can be used to enhance results to verify food. Additionally, technology can be adopted to prevent food from being adulterated or mislabeled. Together, technology can safeguard consumer trust in the brand locally and internationally.

This research reviewed food-related regulations relevant to Australia. While amending and expanding existing laws is an option, it is time-consuming and may not result in reduced food fraud. Instead, an alternate approach to protect consumers is necessary. This research tested regulatory pluralism whereby adopting multiple and varied responses to protect and prevent food fraud. It concludes drawing on a patchwork of traditional and non-traditional, formal and informal responses to protect, monitor and enforce can collectively yield a stronger defense.

The cost of emerging technology may be less financially accessible for some, however drawing on a regulatory pluralism perspective, the downside of not implementing technology designed to protect food may cost businesses and industries catastrophically more through lost revenue, and may disintegrate trust in trade partners as a result of unauthenticated, and potentially vulnerable, products. Through these collective means of control, establishing provenance for all food becomes increasingly possible.

## Data Availability

All data included in this research are publicly available.
